# An exploratory study of Type B variation of the sciatic nerve

**DOI:** 10.3389/fneur.2025.1592879

**Published:** 2025-07-31

**Authors:** Rasyidah Rehir, Jun Mun Teoh, Sumar Chan, Raghad Abdulaziz Almansour, Abeer Saleh Alshaya, Abduelmenem Alashkham

**Affiliations:** 1 Department of Anatomy, Edinburgh Medical School, University of Edinburgh, Edinburgh, United Kingdom; 2 Department of Anatomy, Faculty of Medicine, Universiti Kebangsaan Malaysia, Kuala Lumpur, Malaysia; 3 Department of Anatomy, College of Medicine, King Saud bin Abdulaziz University for Health Sciences, Riyadh, Saudi Arabia; 4 Public Authority for Applied Education and Training (PAAET), Kuwait City, Kuwait; 5 Zawia Faculty of Medicine, University of Zawia, Zawia, Libya

**Keywords:** anatomy, early bifurcation, piriformis, sciatic nerve, variant

## Abstract

**Background:**

Sciatic nerve gives off branches that supply the back of the thigh, leg, and foot. Classically, this nerve emerges from the greater sciatic foramen below the piriformis muscle and subsequently divides into the common fibular and tibial nerves in the distal third of the posterior thigh. However, the course of the sciatic nerve varies among individuals, potentially resulting in nerve compressions. Understanding these variations helps prevent injuries during diagnostic and therapeutic procedures. This study examined the branching patterns of this nerve in the Scottish cadavers using the Beaton and Anson classification, which categorizes them into Type A–G.

**Methods:**

Twelve gluteal regions (4 males, 8 females) with a mean age of 87.3 years, were obtained from a Scottish University regulated by the Human Tissue (Scotland) 2006. The sciatic nerve and its branches were carefully dissected, and the relationship between the nerve and the piriformis muscle was observed and documented.

**Results:**

One left gluteal region of an 89-year-old female (*n* = 1, 8.3%) showed sciatic nerve variation. This variant exhibited early bifurcation, with the common fibular nerve piercing through the piriformis and the tibial nerve passing beneath it (referred to as Type B). The remaining 91.7% of cases, the sciatic nerve exhibited classical presentation (referred to as Type A).

**Conclusion:**

The Type B variation of the sciatic nerve is found in 8.3% of the elderly Scottish cadavers. While it is rare, it is crucial to acknowledge nerve variants to prevent injuries during posterior approach total hip arthroplasty or inadequate sciatic nerve blockade.

## Introduction

The sciatic nerve (SN) is the body’s largest nerve, originating from spinal nerve roots L4 to S3. It innervates the posterior thigh muscles and provides sensory and motor innervation to the skin and muscles of the leg and foot. The nerve exits the pelvis through the greater sciatic foramen, beneath the piriformis muscle (PM), travels through the gluteal region, and enters the posterior thigh. There, it splits into two primary branches: the common fibular nerve (CFN) and the tibial nerve (TN) (1). However, it is important to note that the course and branches of the sciatic nerve can differ among individuals, which can potentially result in nerve compressions. In individuals with an anatomical variation, the sciatic nerve, or parts of it, may exit through or above the piriformis muscle (2). Nerve compressions are more likely to occur at the site where the CFN and TN originate from the pelvis (3).

**Figure 1:**
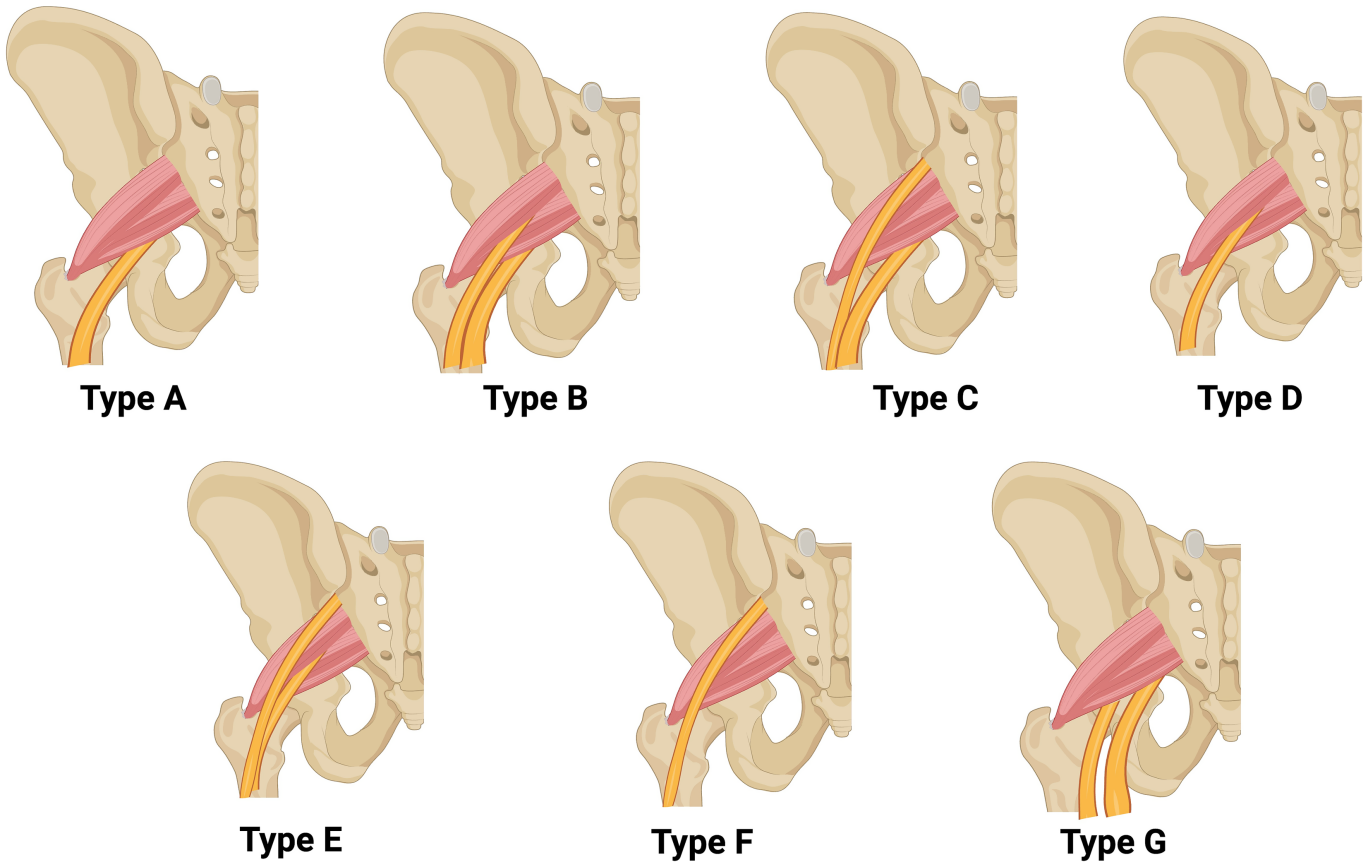
Beaton and Anson classification system of the sciatic nerve as depicted by Type A or Non-Type A (B, C, D, E, F or G variations) (4, 5, 7). This illustration is made by the author using Biorender.com.

Beaton and Anson first classified the relationships between the PM and the SN into six categories and then updated by Tomaszewski which then, became seven categories (4, 5). This classification system is the preferred system for sciatic nerve variations due to its comprehensive detail, clinical relevance, clarity, and simplicity (4, 6). It is widely used in surgical settings, particularly in hip surgery, to help anticipate and mitigate the risks of nerve injury. Anomalous relationships are categorized from type “B” to type “G” (non-Type A), with type “A” representing the normal relationship between the PM and the SN (4, 7) (Table 1; Figure 1). This classification provides a framework for understanding the clinical implications of sciatic nerve variations, which are especially important in conditions such as piriformis syndrome. In 16.9% of patients with piriformis syndrome, there is a variation in the bifurcation of the sciatic nerve (8). Of these, 80.9% are classified as type “B,” 7.6% as type “C,” 3.1% as type “D,” and 0.5% each as types “E” and “F” (8).

**Table 1:** Beaton and Anson’s classification system for the sciatic nerve distinguishes between Type A (normal) and various anomalous variations (B, C, D, E, F, or G) (4, 5, 7).

Types	Description
A	The SN exits medially under the piriformis muscle PM.
B	The SN is pre-bifurcated, with the CFN piercing the muscle belly of the PM.
C	The SN is pre-bifurcated, with the CFN exiting above the PM, and the TN exits medially underneath the PM.
D	The entire SN pierces the PM as a single trunk.
E	The SN is pre-bifurcated, with the CFN exiting above the PM, and the TN piercing the PM.
F	The entire SN exits above the PM as a single trunk.
G	The SN is pre-bifurcated, with both the CFN and TN exiting beneath the PM

SN = sciatic nerve; PM = piriformis muscle; CFN = common fibular nerve; TN = tibial nerve.

The variability in the course of the sciatic nerve can be attributed to its embryonic development, which involves the autonomous formation of the common fibular and tibial divisions (9). The lumbar and sacral plexuses develop at the base of the lower limb bud, forming the primary nerve supply of the lower limb. Nerves from the plexuses differentiate into dorsal and ventral components as they extend into the limb to innervate the respective musculature (10, 11). The common fibular nerve originates from the large dorsal component, whereas the tibial nerve forms from the ventral portion (12). As the subdivisions of the sciatic nerve arise from distinct components, these divisions may separate at varying levels and follow different pathways as they descend to their targets in the lower limb (10, 11).

Interestingly, anatomical variations of the sciatic nerve have been shown to exhibit ethnic and geographical differences (3, 13–19). Both Types A and B variations of the sciatic nerve are observed across all human populations (20). The prevalence of the Type B variation has been reported to range from 1.3 to 33.9% across different studies (3, 14–19, 21).

In relationship to this, non-type A variations in the SN-PM relationship are associated with an increased risk of injury, depending on the clinical approach used (22–25). When anatomical variations of the sciatic nerve are not adequately recognised, they can pose challenges during clinical interventions such as total hip arthroplasty via the posterior approach and sciatic nerve blockade (5, 26, 27). The inability to identify these variations is a common technical error that may result in iatrogenic injury, emphasising the need for a thorough understanding of variant anatomy (28).

Sciatic nerve variants in close relation to the piriformis muscle are at greater risk of intraoperative injury, including stretching, compression and lacerations during total hip arthroplasty (16, 29). Specifically, the location of the common fibular nerve traversing through the piriformis in the Type B variant is highly susceptible to injury from traction, not only during surgery, but also in cases of traumatic hip dislocation (29). Failure to recognise high bifurcation of the sciatic nerve can lead to incomplete sciatic nerve block during popliteal block anaesthesia, as only one subdivision of the nerve may be anaesthetized (17, 30). This variant was also suggested to be more commonly associated with piriformis syndrome due to the entrapment of the common fibular nerve between the piriformis muscle (15). These anomalies are particularly relevant for clinicians performing procedures like imaging-guided injections of the PM, total hip arthroplasty, and piriformis tenotomy for piriformis syndrome. Therefore, the understanding of the prevalence of piriformis and sciatic nerve anomalies is crucial in various clinical contexts. Hence, the aim of this study was to examine the patterns of sciatic nerve branching in the Scottish cadavers.

## Methods

Specimens in this study were obtained from a Scottish Univeristy, regulated by the Human Tissue (Scotland) Act 2006. Ethical approval number ANATED_0036 was given for the use of cadaveric images.

A total of 12 gluteal regions from 6 cadaveric bodies, consisting of two males and four females, with a mean age of 87.3 years. Cadavers with previous trauma or surgery to the gluteal region were excluded. The gluteus maximus muscles of bilateral gluteal regions in each cadaver are dissected and cleaned. The gluteus maximus muscle is then incised along its origin and reflected laterally to expose the underlying gluteus medius, piriformis muscle, gemellus superior, obturator internus, and gemellus inferior muscles along with their neurovascular supply. The fascia covering PM, the sheath covering SN and its branches were carefully dissected. Subsequently, the relationship between the nerve and the piriformis muscle was observed and documented.

Since samples in this study were recruited from a university-based body donation volunteers, this method may introduce sampling bias as the participants are predominantly elderly and from a specific geographic region. These characteristics may limit the generalizability of the results to represent the Scottish population.

## Results

**Figure 2:**
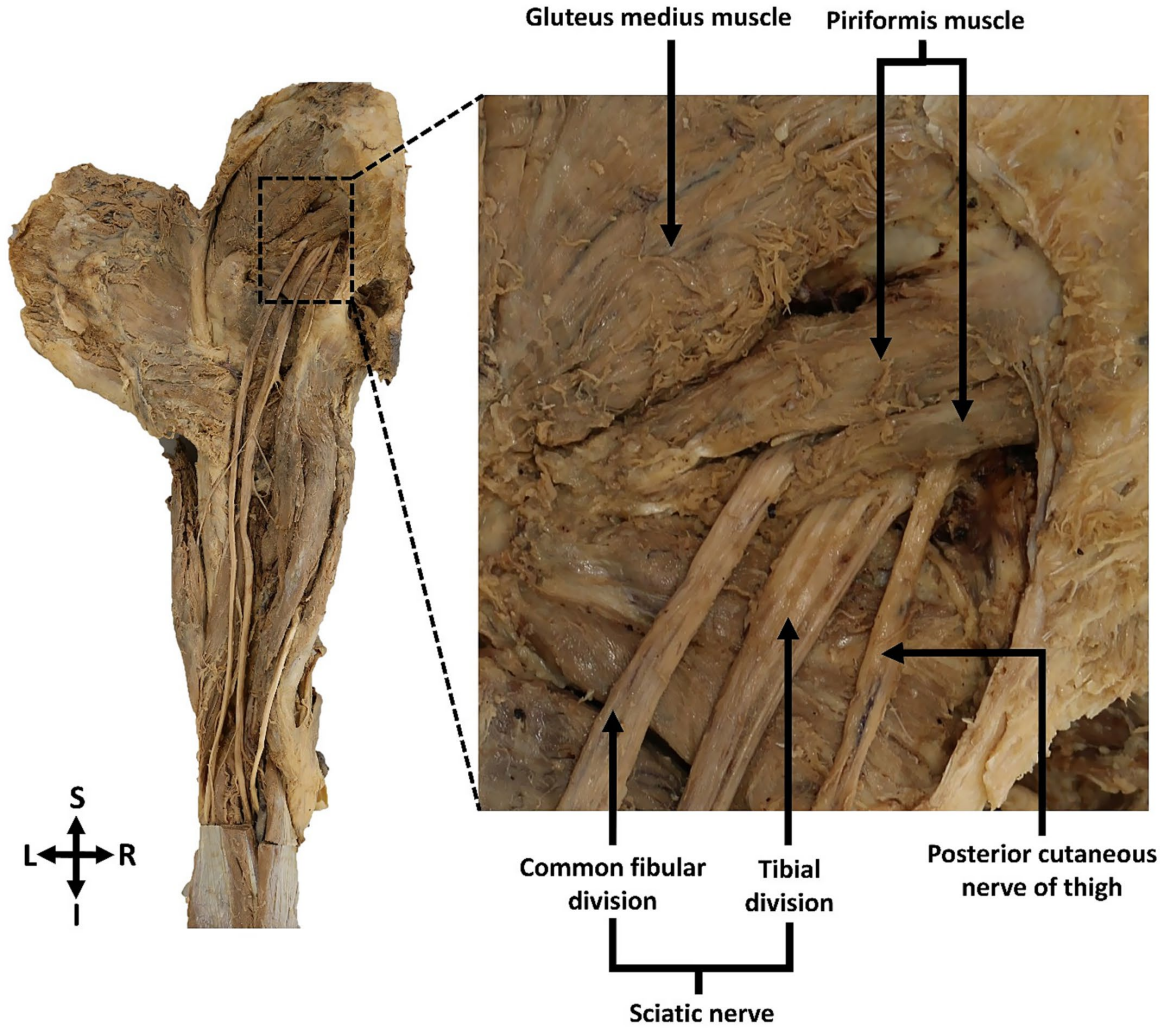
Posterior view of the left gluteal region showing the Type B sciatic nerve variation. The common fibular division of the left sciatic nerve pierced through and exited the piriformis muscle, while the tibial nerve passed inferior to the muscle. The common fibular and tibial nerve divisions remained separate as they proceeded distally to their target muscles in the lower limb.

The anatomical relationships between the sciatic nerve, its branches and the piriformis muscle were documented in twelve dissected gluteal regions. According to Beaton and Anson’s classification (1938b), a Type B variation of the sciatic nerve was identified in 8.3% (n = 1) of the specimens, observed unilaterally in the left gluteal region of an 89-year-old female cadaver. In this case, the left sciatic nerve emerged from the greater sciatic foramen and showed an early bifurcation, with the common fibular nerve piercing through the piriformis muscle, while the tibial nerve emerged below the muscle (Figure 2). Distal to their emergence from the piriformis, the common fibular and tibial nerves followed their typical courses separately to innervate the posterior compartment of the lower limb. In the remaining 91.7% (n = 11) of gluteal regions, the sciatic nerve demonstrated the classical Type A pattern, passing undivided inferior to the piriformis muscle. No other variation types were observed in this sample.

## Discussion

This exploratory study documented the variation in the sciatic nerve and its prevalence within the cadaveric population at the University of Edinburgh, highlighting the significance of such anatomical variations and their implications for clinical practice. In comparison with prevalence data from a meta-analysis, Type A sciatic nerve presentation is the most common, with a reported pooled prevalence of 90% (8), closely aligning with the 91.7% observed in this study. The Type B variant, which is characterised by the sciatic nerve subdivisions passing between and below the piriformis muscle, exhibited an 8.3% prevalence in this sample, consistent with the previous pooled prevalence of 8% (8). The course and bifurcation pattern of sciatic nerve showed great variability as categorised by Beaton and Anson (4). The nerve subdivisions emerged above and below the piriformis muscle in Type C whereas the undivided nerve pierced through the muscle in Type D. In contrast to previously reported cases (8), Types C, D, E, F and G were absent in the current study.

The findings of this study were also in line with the previous reports on sciatic nerve variation across different demographical and geographical backgrounds. In this study, the prevalence of the Type B variation is present in 10% of the Scottish cadavers, which lies within the prevalence range of 1.3 to 33.9% across different studies (3, 14–19, 21). The wide range of reported prevalence values (1.3–33.9%) suggests that ethnic differences may influence sciatic nerve variations. Notably, the prevalence is significantly higher in East Asia (24%) compared to Europe (9%) (8), where the Scottish cadavers from this study can be classified. Contrary to previous studies indicating no significant differences in the laterality of sciatic nerve variations (8), the Type B variant in this study was observed exclusively in the left gluteal region of a female cadaver. Similar to observations in this study, sciatic nerve variations were more frequently identified in females compared to males (29, 31, 32). A previous meta-analysis found that the Type B variant was twice as common in females as in males, though this difference was not statistically significant (8). This gender-based prevalence disparity remains unclear, but it has been suggested that the development of the sciatic nerve close to the female reproductive organs may play a role (8).

Differences in the embryological development timing of the sciatic nerve and piriformis muscle may contribute to variations in their anatomical relationship (8). Formation of the sciatic nerve begins approximately the sixth week of embryonic development, while the piriformis muscle develops by the eighth week (33). This discrepancy in developmental timing and speed may contribute to variations in the anatomical relationship between the two structures before the definitive insertion of piriformis forms at around 15 weeks (33). The formation of piriformis muscle from two separate myotome structures may also allow the common fibular nerve to traverse the muscle before these myotomes fuse into a single structure (34, 35), leading to the Type B variation. While direct genetic studies specifically linking genetic factors to sciatic nerve variations are currently limited, Kasapuram et al. (34) proposed that nerve pathway morphology may be influenced by the expression levels of molecules which regulate nerve growth and direction, such as netrins, slits, semaphorins and ephrins (36). Genetic predisposition resulting in these molecular changes could contribute to population-based differences in sciatic nerve anatomy. Further genetic studies are needed to establish a direct association.

Awareness of sciatic nerve variations not only improves treatment outcomes, but also aids in the diagnosis of related pathologies. While sciatic nerve variations do not always correlate to piriformis syndrome, they are potential causes of this condition, leading to pain in the gluteal region (2, 37). Although sciatica commonly results from spinal degenerative disc disorders, nerve entrapment in piriformis syndrome can mimic sciatica symptoms and has been reported to account for 6–8% of sciatica cases (38, 39). Therefore, recognising sciatic nerve variants is crucial for accurately diagnosing associated pathologies to ensure appropriate treatment is administered (40, 41). The Type B variant was previously suggested to be more commonly associated with piriformis syndrome due to the entrapment of the common fibular nerve between the piriformis muscle (15). Although sciatica commonly results from spinal degenerative disc disorders, nerve entrapment in piriformis syndrome can mimic sciatica symptoms and has been reported to account for 6–8% of sciatica cases (38, 39).

This study presented several limitations, including small sample size, which restricted the examination of other sciatic nerve variations, specific variant laterality, age, gender prevalence, and also sampling bias. Furthermore, the absence of the donor’s medical histories did not allow investigation into potential correlations between anatomical variations of sciatic nerve and clinical presentations. Despite these limitations, the sciatic nerve variant observed in the current study highlighted the importance of an in-depth understanding of anatomical variability and the need for future research into how such variations impact clinical practice and outcomes. Future studies should also aim to include a more diverse sample by recruiting from multiple locations and using random sampling methods to enhance external validity.

In conclusion, variation in the sciatic nerve anatomy is present in 8.3% of the Scottish elderly cadaveric population. In this case, the left sciatic nerve exhibited early bifurcation, with the common fibular nerve piercing through piriformis and the tibial nerve passing beneath it (referred to as Type B). In the remaining 91.7% of cases, the sciatic nerve exhibits the classical presentation (referred to as Type A). The Type B variation of the sciatic nerve is found in 8.3% of the elderly Scottish cadavers. Although anatomical variations of the sciatic nerve are relatively uncommon, it is essential for medical practitioners to understand such variants and consider preoperative imaging for high-risk patients to prevent injuries during medical procedures. Future studies using imaging techniques in living patients, or genetic research would be most beneficial and should be conducted on a larger scale to comprehensively investigate the prevalence of sciatic nerve variations among the Scottish population.

## Data Availability

The original contributions presented in the study are included in the article/supplementary material, further inquiries can be directed to the corresponding author.
