# Correction: An exploratory study of Type B variation of the sciatic nerve

**DOI:** 10.3389/fneur.2026.1900196

**Published:** 2026-06-19

**Authors:** Rasyidah Rehir, Jun Mun Teoh, Sumar Chan, Raghad Abdulaziz Almansour, Abeer Saleh Alshaya, Abduelmenem Alashkham

**Affiliations:** 1 Department of Anatomy, Edinburgh Medical School, University of Edinburgh, Edinburgh, United Kingdom; 2 Department of Anatomy, Faculty of Medicine, Universiti Kebangsaan Malaysia, Kuala Lumpur, Malaysia; 3 Department of Anatomy, College of Medicine, King Saud bin Abdulaziz University for Health Sciences, Riyadh, Saudi Arabia; 4 Public Authority for Applied Education and Training (PAAET), Kuwait City, Kuwait; 5 Zawia Faculty of Medicine, University of Zawia, Zawia, Libya

**Keywords:** anatomy, early bifurcation, piriformis, sciatic nerve, variant

In the published article, **Affiliation** “Public Authority for Applied Education and Training (PAAET), Kuwait” was omitted for author “Abeer Saleh Alshaya.” This affiliation has now been added for “Abeer Saleh Alshaya.”

**Affiliation** “Zawia Faculty of Medicine, University of Zawia, Zawia, Libya” was omitted for author “Abduelmenem Alashkham.” This affiliation has now been added for author “Abduelmenem Alashkham.”

**Affiliation** “Department of Anatomy, Edinburgh Medical School, University of Edinburgh, Edinburgh, United Kingdom” was erroneously given as “Department of Anatomy, Edinburgh Medical School: Biomedical Sciences, University of Edinburgh, Edinburgh, United Kingdom.”

The original version of this article has been updated.

